# Identification of 5-methylcytosine-related signature for predicting prognosis in ovarian cancer

**DOI:** 10.1186/s40659-021-00340-8

**Published:** 2021-06-29

**Authors:** Lei Wang, Song Gao

**Affiliations:** Department of Obstetrics and Gynecology, Shengjing Hospital of China Medical University, Shenyang, 110014 Liaoning China

**Keywords:** Ovarian cancer, 5-methylcytosine, Risk score, Molecular subtypes

## Abstract

**Background:**

Ovarian cancer is one of the most common malignancies often resulting in a poor prognosis. 5-methylcytosine (m5C) is a common epigenetic modification with roles in eukaryotes. However, the expression and function of m5C regulatory factors in ovarian cancer remained unclear.

**Results:**

Two molecular subtypes with different prognostic and clinicopathological features were identified based on m5C regulatory factors. Meanwhile, functional annotation showed that in the two subtypes, 452 differentially expressed genes were significantly related to the malignant progression of ovarian cancer. Subsequently, four m5C genes were screened to construct a risk marker predictive of overall survival and indicative of clinicopathological features of ovarian cancer, also the robustness of the risk marker was verified in external dataset and internal validation set. multifactorial cox regression analysis and nomogram demonstrated that risk score was an independent prognostic factor for ovarian cancer prognosis.

**Conclusion:**

In conclusion, our results revealed that m5C-related genes play a critical role in tumor progression in ovarian cancer. Further detection of m5C methylation could provide a novel targeted therapy for treating ovarian cancer.

**Supplementary Information:**

The online version contains supplementary material available at 10.1186/s40659-021-00340-8.

## Background

As a malignant tumor seriously threatening women’s health, ovarian cancer has the third highest incidence among all malignant tumors of the female reproductive system, with a mortality rate ranking the first highest [1]. According to the statistics, 2,39,000 new cases (3.6% of all cancer cases) of ovarian cancer are diagnosed, resulting in 1,52,000 deaths (4.3% of all cancer deaths) each year [2]. Due to a lack of effective screening strategies, ovarian cancer shows a late onset of clinical symptoms and is prone to widespread pelvic and abdominal implantation and dissemination, therefore approximately 60% of ovarian cancer patients are diagnosed at an advanced stage [3]. Although surgery, chemotherapy, biological therapy and gene therapy are widely applied in the treatment of ovarian cancer [4], the 5-year survival rate for ovarian cancer patients still remains as low as 35–38% [5]. Therefore, exploring the mechanism of ovarian cancer is important for the early detection, diagnosis and treatment of ovarian cancer [6–8].

Epigenetic modifications, which mainly include DNA methylation and histone modifications, are chemical alterations in nucleic acids that do not change DNA sequence but play a key role in genetics, growth, longevity, aging and diseases [9, 10]. DNA 5-methylcytosine (m5C), which is the most abundant DNA modification in mammalian cells, is characterized by the addition of a methyl group to the carbon-5 position of cytosine base [11]. In recent years, knowledge of RNA modifications has been greatly expanded from fine-tuned chemical structural features of non-protein-coding RNAs to dynamically regulated, reversible, post-transcriptional regulators that are widely present in a variety of cellular processes [12]. Mammalian RNA methylation modifications mainly include N6-methyladenosine (m6A), N1-methyladenosine (m1A), pseudouridine (Ψ) and m5C, but previous studies on RNA methylation mainly focuse on m6A [12, 13]. However, new evidence gradually revealed the role in m5C in post-transcriptional regulation.

5-methylcytosine methylation involves a range of regulators, including m5C methyltransferases, demethylases and “readers”. The methyltransferase “writer” complex increases methylation at the C5 position of the RNA, and then a different “reader” protein recognizes and binds the methylated mRNA, while the “eraser” protein reverses the m5C modification by degrading the written methylation [14]. In addition, to date, m5C modification has been shown to play a key role in the pathogenesis of bladder cancer [15], hepatocellular carcinoma (HCC) [16], glioblastoma multiforme (GBM) [17] and leukemia [18]. The above results suggest a promising function of m5C modification in cancer therapy. However, specific genetic signature and prognostic significance of m5C-related regulators in ovarian cancer remained to be discovered.

**Fig. 1:**
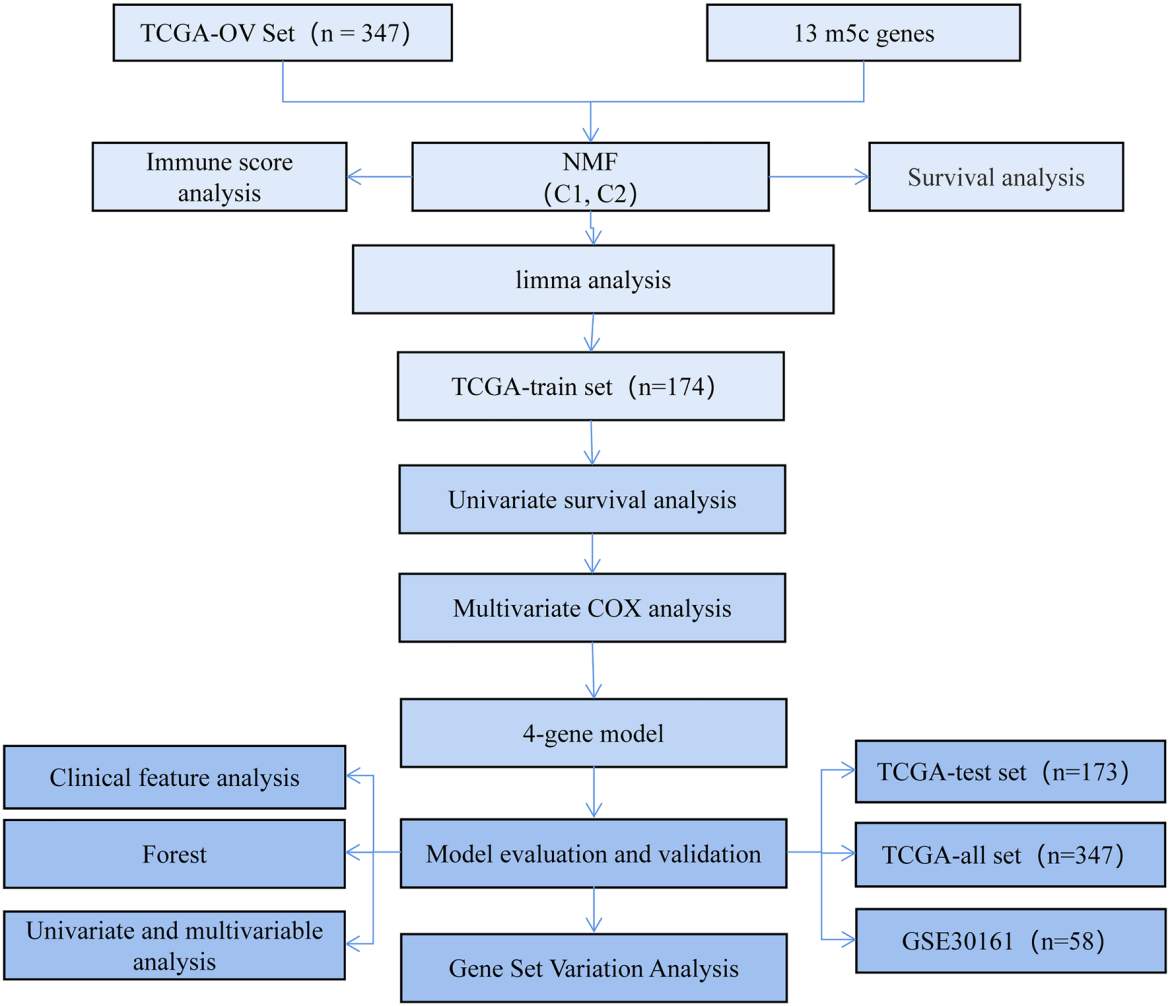
Work flow chart

In the present study, two molecular subtypes of ovarian cancer were identified from TCGA ovarian cancer samples based on 13 m5C-regulated genes, which were found to be closely associated with clinicopathological features. In addition, the changes observed in m5C regulatory genes were significantly associated with a higher tumor stage. Based on the least absolute shrinkage and selection operator (LASSO) and multivariate cox regression model, we constructed a gene signature of m5C regulators to effectively predict the prognosis of ovarian cancer patients. In conclusion, we detected changes in m5c-related genes capable of affecting some key regulatory molecules, which contribute to ovarian cancer progression (Fig. 1).

## Results

### Non-negative matrix factorization analysis of m5C-regulatory genes

**Fig. 2:**
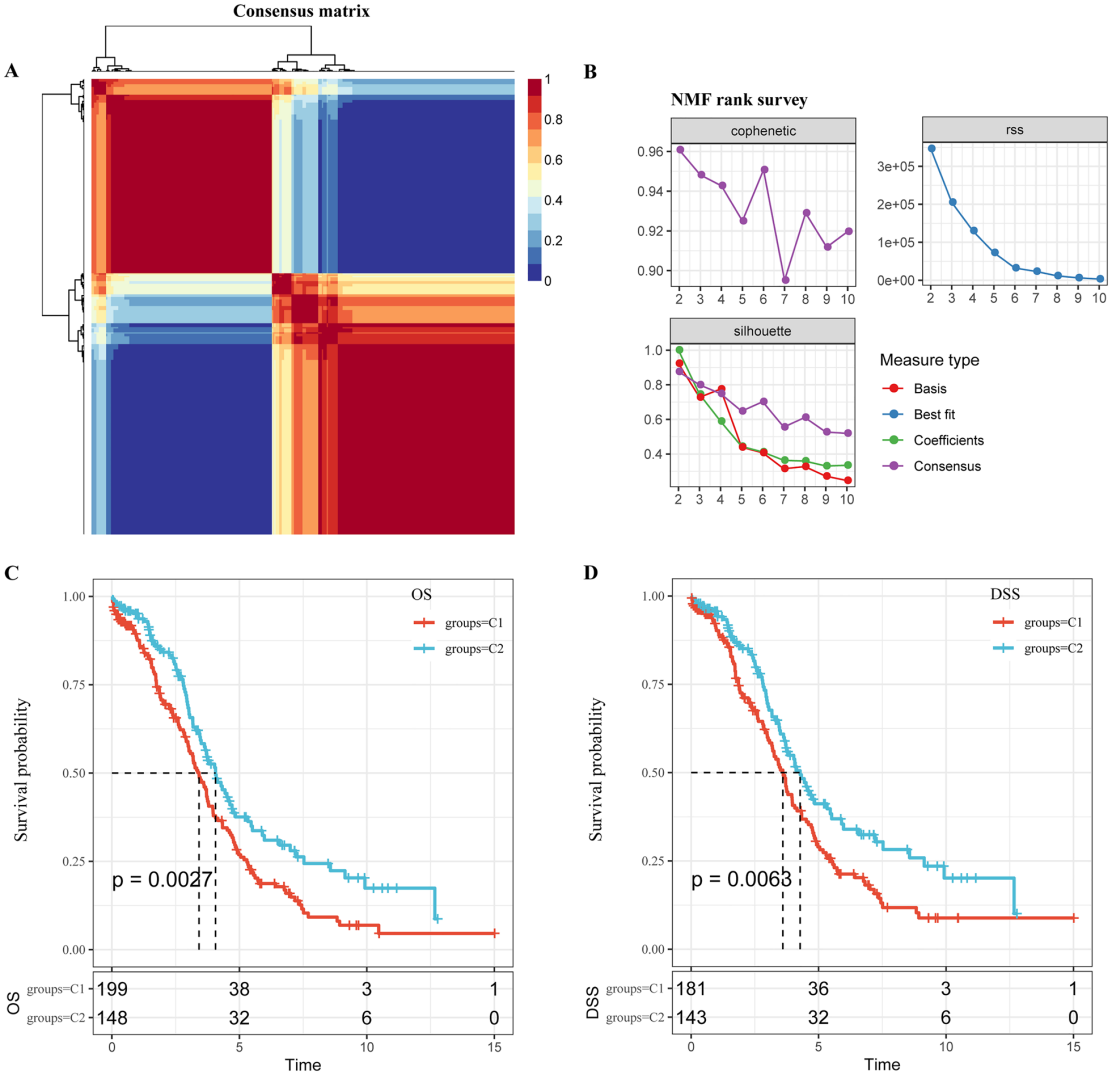
Non-negative matrix factorization analysis of m5C-regulatory genes. **A** Non-negative matrix factorization analysis was conducted to identify two subtypes, namely C1 and C2. **B** Three index, cophenetic, dispersion and silhouette. **C** Significant differences was observed in overall survival (OS) and disease-specific survival (DSS) between the two groups. **D** Significant differences was observed in disease-specific survival (DSS) between the two groups

Based on the 13 identified m5C-regulatory gene expression profiles from 347 patients with ovarian cancer in the TCGA dataset, non-negative matrix factorization analysis was conducted to identify two subtypes, namely, C1 and C2 (Fig. 2A), according to three index, cophenetic, dispersion and silhouette (Fig. 2B). Moreover, we found significant differences in overall survival (OS) and disease-specific survival (DSS) between the two groups (p  =  0.0027 and p  =  0.0063; Fig. 2C, D). The survival of patients in the C1 subtype was obviously shorter than those in the C2 subtypes. These results indicated that consensus clustering of m5C regulatory factors could identify ovarian cancer subtypes with different prognosis.

### The interrelation of m5C-associated molecular subtypes and clinicopathological characteristics of patients with ovarian cancer

**Fig. 3:**
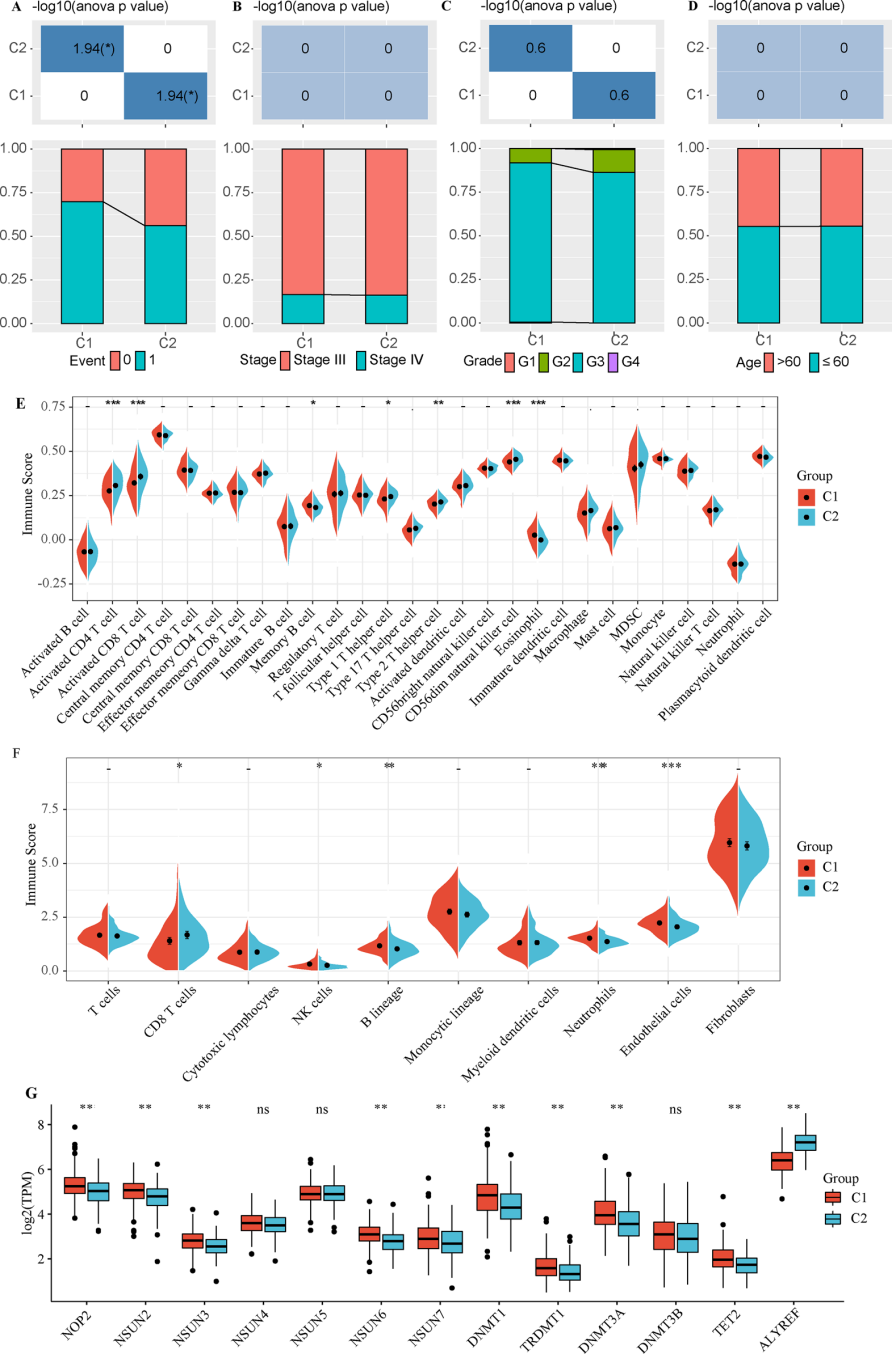
The interrelation of m5C-associated molecular subtypes and clinicopathological characteristics in patients with ovarian cancer. **A** The death rate of the C1 subgroup with poor prognosis was higher. **B**–**D** There was no significant difference in stage, grade and age between the two subtypes. **E**, **F** C1 was significantly higher than C2 in memory B cell and eosinophil immune-related cells, and lower than C2 in activated CD4 T cell, activated CD8 T cell, type 1 T helper cell, type 2 T helper cell and CD56dim natural killer cell. **G** the expression of 13 M5C related genes in the two subtypes

We compared the distribution of different clinical features in the two molecular subtypes, and determined whether the clinical features were different in different subtypes. The results showed that the death rate of the C1 subgroup with poor prognosis was higher (Fig. 3A). There was no significant difference in stage, grade or age between the two subtypes (Fig. 3B–D).

The immune cell score between the two molecular subtypes showed that memory B cells and eosinophil immune-related cells were significantly higher in C1 than C2, and that activated CD4 T cells, activated CD8 T cells, type 1 T helper cells, type 2 T helper cells and CD56dim natural killer cell were lower in C1 than C2 (Fig. 3E, F). At the same time, the expression of 13 M5C related genes in the two subtypes were analyzed. In addition to NSUN4, NSUN5 and DNMT3B genes, the remaining 9 genes were significantly differentially expressed in the two subtypes (Fig. 3G).

### Construction of the risk score signature by four m5C-regulatory genes

**Fig. 4:**
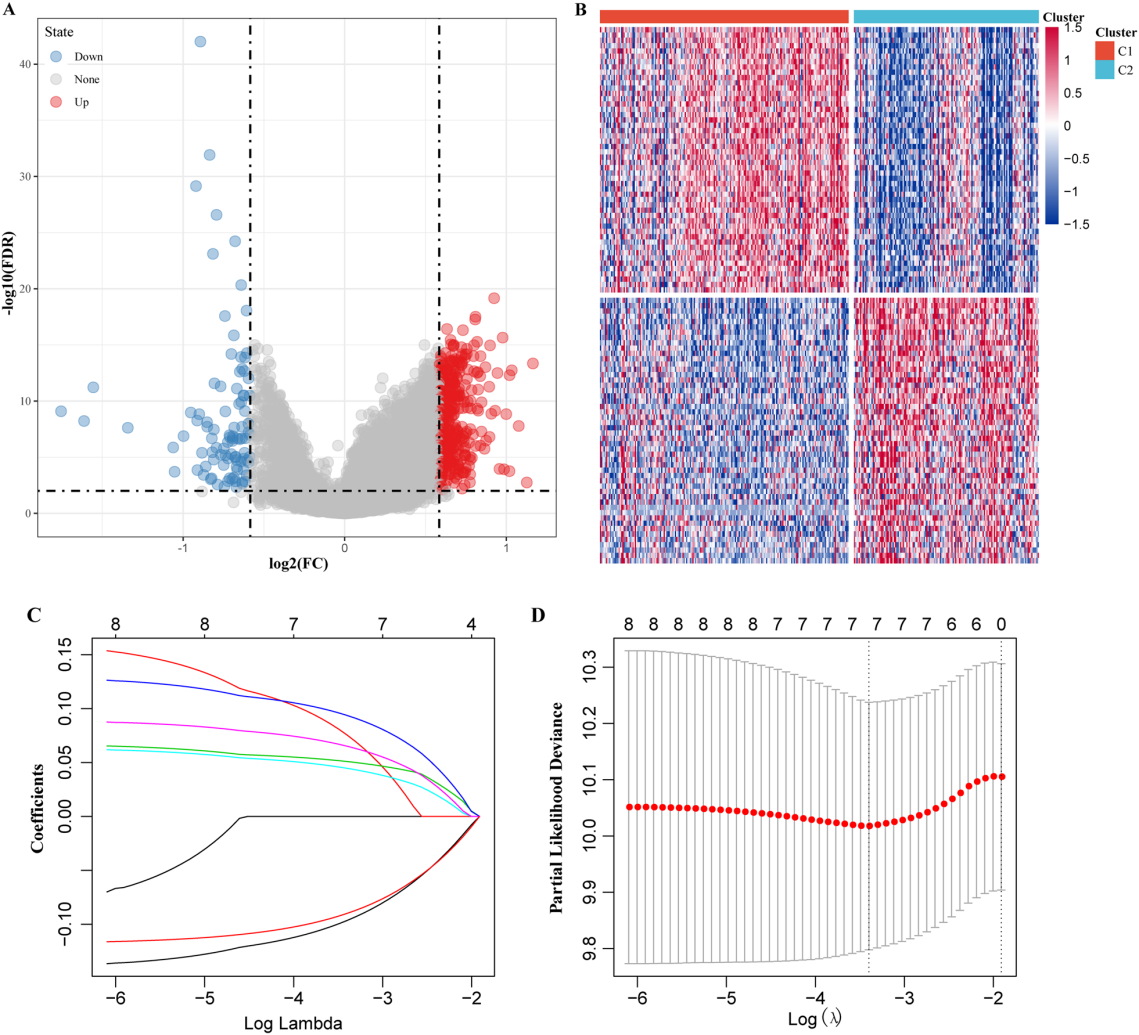
Construction of the risk score signature by four m5C-regulatory genes. **A** Volcano plot of differentially expressed genes. **B** Heatmap of differentially expressed genes. **C** The change trajectory of each independent variable, the horizontal axis represents the log value of the independent variable lambda, and the vertical axis represents the coefficient of the independent variable; **D** the confidence interval under each lambda

Firstly, the differential expression of genes between molecular subtypes was determined using the limma package. According to the threshold FDR  <  0.05 and |log2FC| >  log2(1.5), 354 up-regulated and 98 down-regulated genes were identified (Fig. 4A, B).

Furthermore, KEGG pathway analysis and GO functional enrichment analysis of differentially expressed genes were performed using the R software package WebGestaltR (V0.4.2). The GO function annotation showed 99 items with significant difference from CC (Additional file 1: Figure S1A) and 57 items with significant difference from MF (Additional file 1: Figure S1B). The KEGG pathway was enriched to 19 significant pathways (Additional file 1: Figure S1C), including the Notch signaling pathway, ECM-receptor interaction, focal expression and PI3K-Akt signaling pathway and other tumor-related pathways.

We further explored the performance of 452 genes in predicting ovarian cancer prognosis through univariate survival analysis using cox proportional hazards models based on expression levels in the TCGA training dataset, and here we obtained eight genes associated with prognosis. To improve the robustness of the eight m5C-regulatory genes, these genes were subjected to the least absolute shrinkage and selection operator (LASSO) cox regression algorithm in the TCGA training dataset (Fig. 4C, D). Four m5C-regulatory genes were screened to construct a risk score signature, a the formula for the risk score was as follow:

$${\text{RiskScore }} = {\text{ 0}}{\text{.1452663}}^{ * } {\text{FCGBP }} + {\text{ 0}}{\text{.1300001}}^{ * } {\text{HOXB}} - {\text{0}}{\text{.1675289}}^{ * } {\text{TYMSO}} - {\text{0}}{\text{.1130089}}^{ * } {\text{CLDN10}}{\text{.}}$$

To better understand the role of these four prognostic genes in ovarian cancer, K-M analysis was performed both in the TCGA training dataset, in which samples were classified by high or low expression according to the median gene expression level. FCGBP and CLDN10 genes were found to be significantly correlated with OS (Additional file 2: Figure S2).

**Fig. 5:**
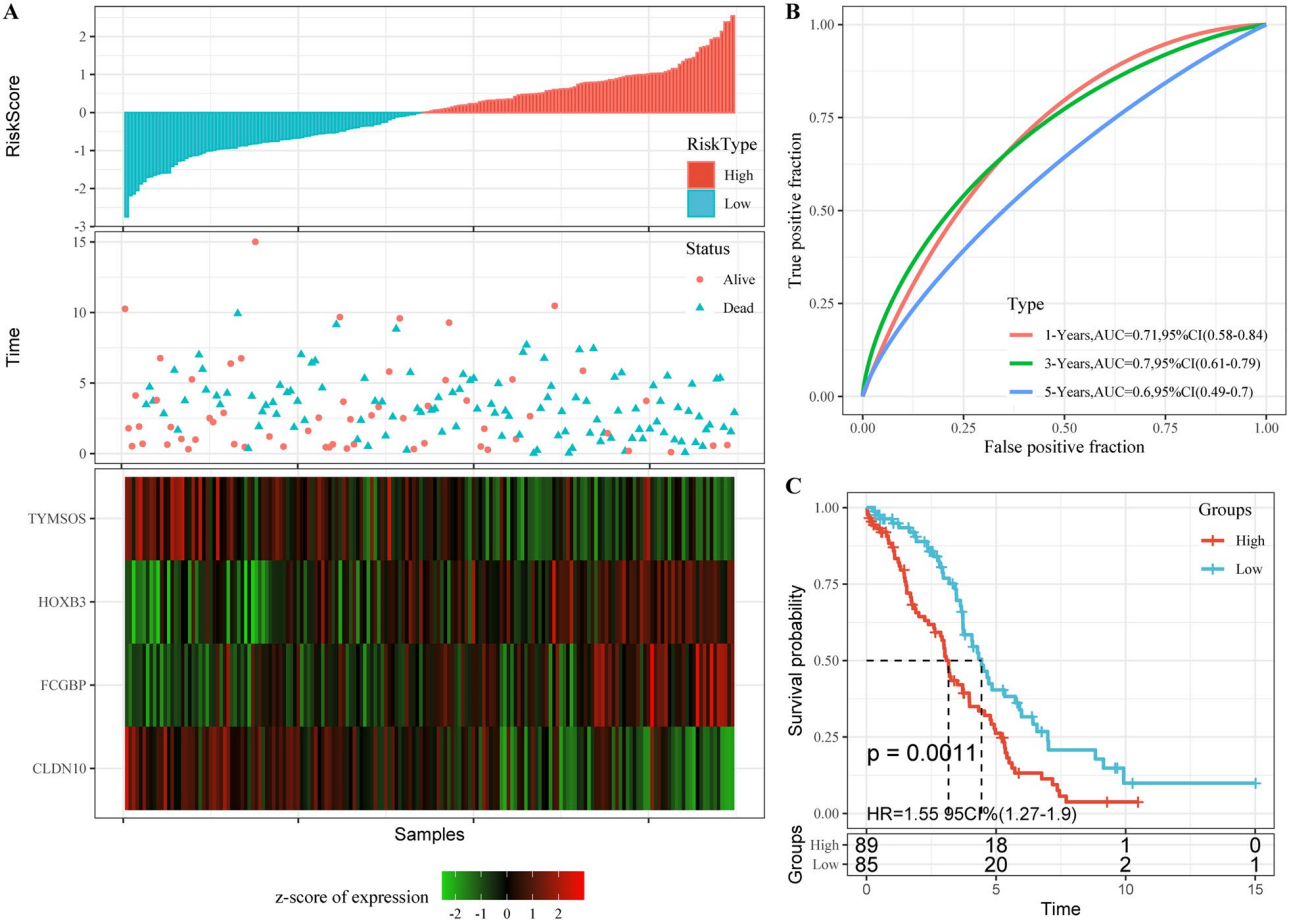
Predictive effect of the risk score. **A** RiskScore, survival time and survival status and 4 mRNAs expression of each samples in TCGA training set. **B** Classification ROC curve and AUC of 4-mRNA signature in TCGA training set. **C** KM survival curve of 4-mRNA signature in the TCGA training set

To evaluate the performance of the risk score signature in predicting the clinical outcomes of ovarian cancer patients, and the median score of all patients’ scores was used as a standard to divide the data in the TCGA training dataset into high- and low-risk groups. Our analyses indicated that the number of patients who died increased significantly as the risk score increased, and that FCGBP and HOXB3 acted as risk factors, while TYMSOS and CLDN10 were protective factors (Fig. 5A). The area under the ROC curve (AUC) of prognostic risk scores for 1-, 3-, and 5-year were 0.71, 0.7 and 0.6, respectively (Fig. 5B). In addition, there was a significant difference in overall survival between the high-risk group and low-risk group (p  =  0.0011; Fig. 5C).

### The robustness of the prognostic of the risk score signature in predicting ovarian cancer prognosis

**Fig. 6:**
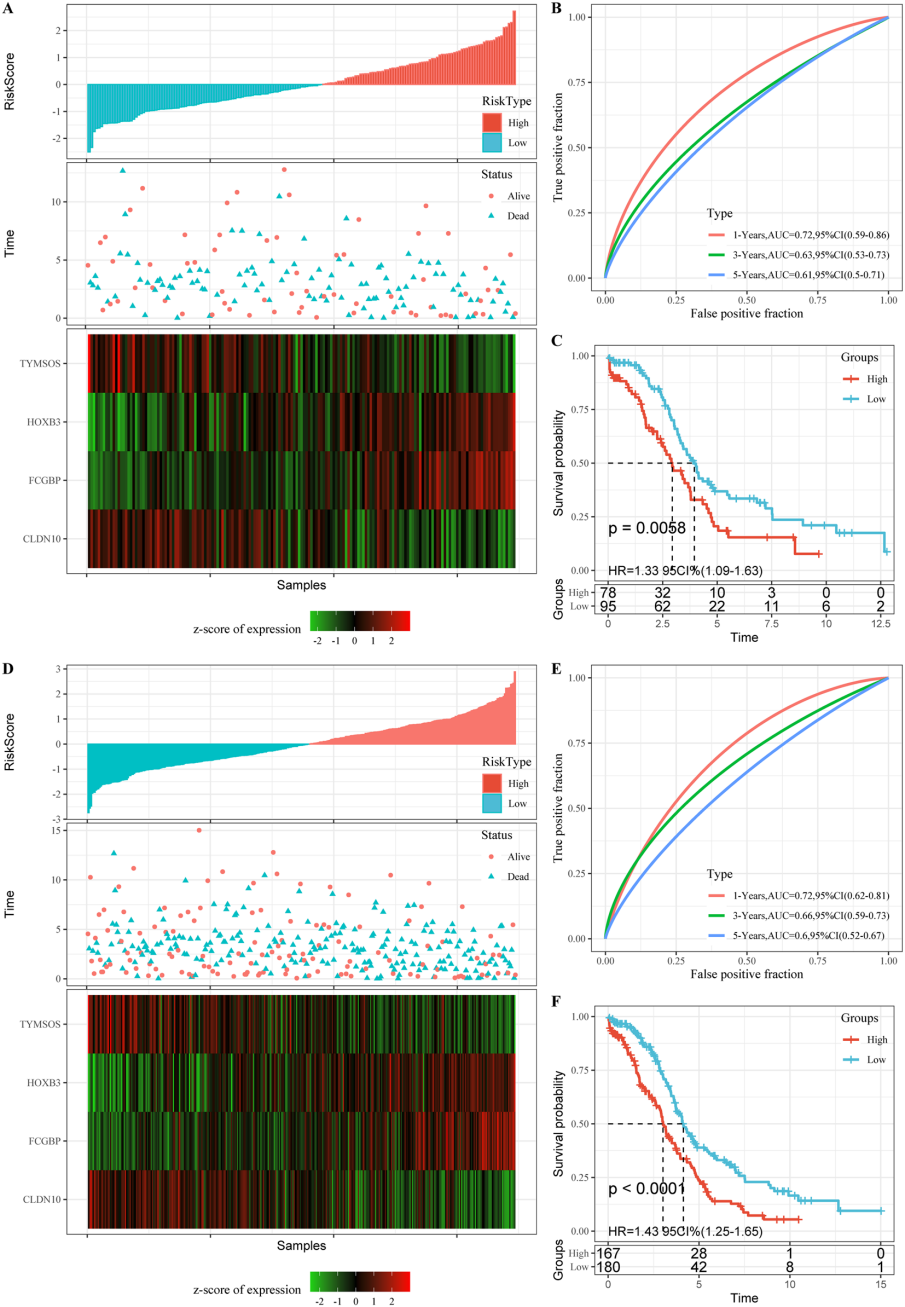
Robustness of risk models. **A** RiskScore, survival time and survival status and 4 mRNAs expression of each samples in TCGA test dataset. **B** Classification ROC curve and AUC of 5-miRNA signature in TCGA test dataset. **C** KM survival curve of 4-mRNA signature in the TCGA test dataset. **D** RiskScore, survival time and survival status and 4 mRNAs expression of each samples in entire TCGA dataset. **E** Classification ROC curve and AUC of 4-mRNA signature in entire TCGA dataset. **F** KM survival curve of 5-miRNA signature in the entire TCGA dataset

To examine the robustness of the risk score signature in clinical outcomes of ovarian cancer patients, TCGA test dataset and entire TCGA dataset samples were assigned into high- and low-risk groups. The analyses indicated that the number of patients who died increased significantly as the risk score increased, and that FCGBP and HOXB3 acted as risk factors, while TYMSOS and CLDN10 were protective factors (Fig. 6A, D). The area under the ROC curve (AUC) of prognostic risk scores for 1-, 3-, and 5-year were also higher (Fig. 6B, E). In addition, there was also a significant difference in overall survival between the high-risk group and low-risk group (p  =  0.0011; Fig. 6C, F).

Multivariate cox survival analysis was performed in the external validation dataset GSE30161 using the same genes as in the training set model, and then the RiskScore of each sample was calculated separately according to the expression level of the samples to plot RiskScore distribution of the samples. The proportion of deaths in samples with high RiskScore was significantly higher than that in samples with low RiskScore, which was consistent with the predicting performance on the TCGA training set (Additional file 3: Figure S3A). The area under the ROC curve (AUC) of prognostic risk scores for 1-, 3-, and 5-years were 0.63, 0.74 and 0.75, respectively (Additional file 3: Figure S3B). In addition, there was a significant difference in overall survival between the high-risk group and low-risk group (p  =  0.0016; Additional file 2: Figure S3C). From the comprehensive analyses above, it could be concluded that the prognostic predicting performance of the risk score was accurate and stable.

### Risk model and clinical feature prognostic analysis

**Fig. 7:**
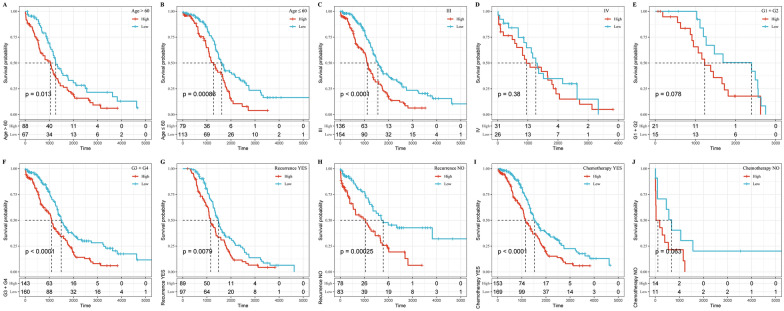
Risk model and clinical features prognostic analysis

We also found that the 4-gene signature could significantly differentiate the high- and low-risk groups by age, Stage III, G3  +  G4, recurrence or not, and chemotherapy samples (Fig. 7; p  <  0.05), further indicating that the model still had an equally strong predictive power across different clinical characteristics.

**Fig. 8:**
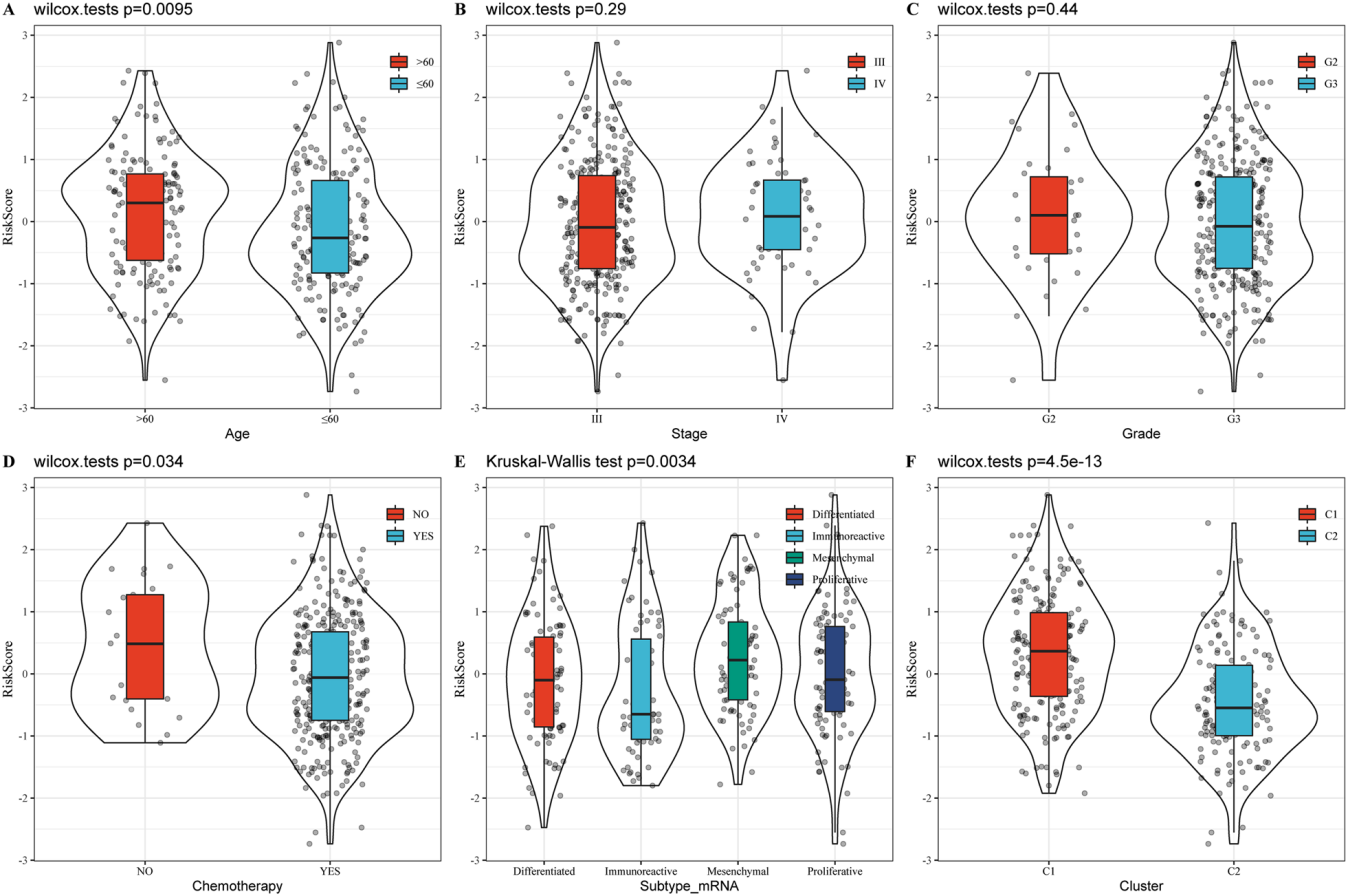
The distribution of the RiskScore among the clinical characteristic subgroups

The distribution of the RiskScore among the clinical characteristic subgroups was compared, and there was no significant difference in the RiskScore in the stage and grade subgroups (Fig. 8B, C; p  > 0.05). RiskScore showed a significant difference between age group and chemotherapy group (Fig. 8; p  <  0.05). At the same time, after comparing the difference of risk scores in molecular subtypes, it was observed that the risk score of C1 subtype with poor prognosis was significantly higher than that of C2 molecular subtype with good prognosis. The difference between the existing molecular subtypes Risk scores also had significant differences (Fig. 8B, C; p  < 0.05).

### Independent prognostic factor evaluation and correlation with clinical characteristics

**Fig. 9:**
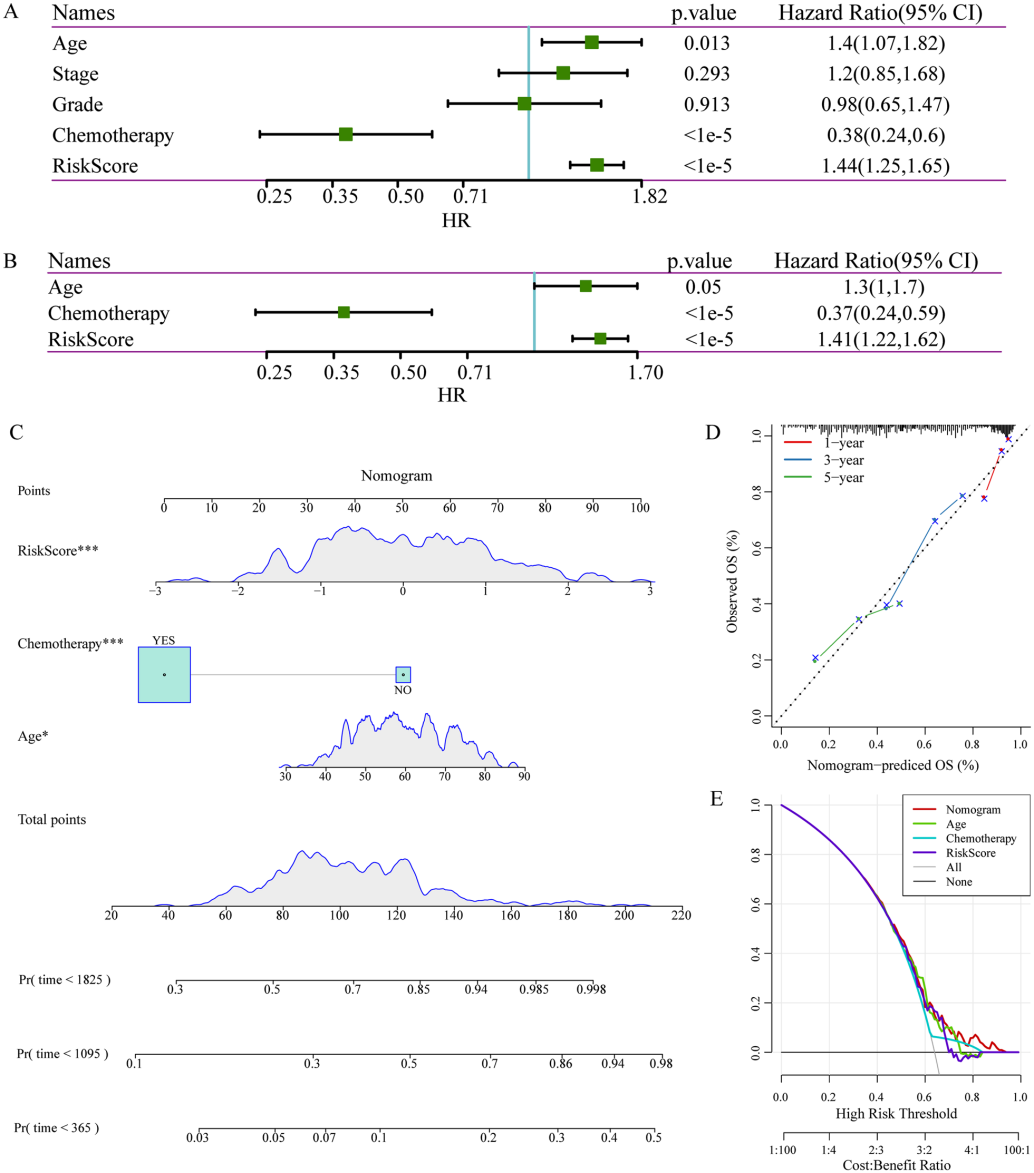
Independent prognostic factor evaluation and correlation with clinical characteristics. **A** Results of univariate analysis of clinical characteristics and RiskScore. **B** Results of multivariate analysis of clinical characteristics and RiskScore. **C** A nomogram constructed by RiskScore and clinical features; **D** A correction chart for survival rate of the nomogram; **E** DCA curve

We also investigated whether this risk score was an independent prognostic factor based on four clinicopathological features. Univariate and multivariate cox regression analyses were performed with the TCGA dataset. It was found that the risk score, age, and chemotherapy were significantly correlated with prognosis using the univariate analysis (Fig. 9A). Multivariate analysis based on the above factors was performed, the data showed that risk score, age, and chemotherapy were strongly associated with the OS (Fig. 9B). The consensus results demonstrated that the risk score constructed by the four m5C-regulatory genes was an effective and independent prognostic factor for predicting ovarian cancer outcome.

We next developed a prognostic nomogram to examine the relationship between clinical traits and RiskScore in TCGA cohort, and found that RiskScore characteristics showed the greatest impact on survival prediction (Fig. 9C). The prediction accuracy of the calibration curve evaluation model showed that the predicted calibration curves for the three calibration points at 1, 3 and 5 years were close to coinciding with the standard curve (Fig. 9D), which suggested that the model had a high prediction performance. In addition, after evaluating the reliability of the model using DCA (decision curve), it was found that the accuracy of RiskScore and nomogram were significantly higher than the extreme curves, specifically, the nomogram was higher than RiskScore, and age and chemotherapy were close to the extreme curves (Fig. 9E). This indicated that both RiskScore and nomogram were highly reliable. In addition, in order to compare prognostic differences among pathological subtypes of ovarian cancer, we evaluated the classification performance of high-grade and low-grade serous ovarian cancer using subtype-specific genes. Specifically, we selected GSE140082 as the validation set, and the GEO data set included 211 cases of high-grade serous ovarian cancer and 63 cases of low-grade serous ovarian cancer. Based on the risk model, we calculate the risk scores of high-grade serous carcinoma and low-grade serous carcinoma respectively, and divide the patients into low-risk groups. The results show that there is a significant difference between high- and low-risk group in the prognosis of high-grade serous carcinoma patients (Additional file 4: Figure S4A–C). In low-grade serous carcinoma, the expression trend of the four characteristic genes is consistent with expression of TCGA, but the prognosis is not significant (Additional file 4: Figure S4D–F). These results suggest the existence of different molecular features for different pathological subtypes, and the identification of a new molecular staging of high-grade serous ovarian cancer based on m5c-related genes is a new molecular staging that is distinct from and can be complementary to the traditional clinical staging.

## Discussion

The important role of aberrant RNA epigenetic modifications in tumorigenesis, progression, and patient prognosis has been increasingly confirmed, pointing to a potential application of epigenetic modifiers in the diagnosis and prognosis of ovarian cancer. In the past, a large number of reports on ovarian cancer and 5-methylcytosine have focused on DNA methylation, which is considered as an ideal diagnostic biomarker for tumors [19–21]. Specifically, compared with normal ovarian tissues, a significant promotion in 5-mC expression in EOC is correlated with pathologic stage, tumor grading, lymph node metastasis, and vascular thrombosis [19]. This study focused on RNA epigenetic modification, and by detecting the abnormal expression of m5C-related regulatory factors, m5C was found to be involved in the occurrence and development of ovarian cancer and was related to the cancer prognosis. By analyzing the expression profiles of m5C regulatory factors from two open databases (TCGA and GEO databases), two subtypes with different clinicopathological characteristics and prognosis were identified. In addition, the subtypes were closely related to tumor-related clinical features and immune infiltration. According to the differentially expressed genes between the molecular subtypes, a related risk scoring algorithm was constructed to divide ovarian cancer patients into high-risk groups and low-risk groups, and could accurately predict the clinical outcome of ovarian cancer patients. In addition, univariate and multivariate cox regression analysis showed that RiskScore was an independent prognostic factor for patients with ovarian cancer. The four m5C regulatory factors can be used as an effective prognostic marker to stratify ovarian cancer patients according to risk score, providing new insights for targeted therapy.

The four m5C regulators have been previously reported to be involved in the progression of malignant tumors. According to the latest literature, study revealed that FCGBP participates in the development of gastric neoplasm [22], and is also a key regulatory factor in the epithelial-mesenchymal transformation process of gallbladder cancer metastasis and prognosis [23]. Recently, a paper also showed that FCGBP is high-expressed in ovarian cancer in GSE12470 and GSE40595, and high-expressed FCGBP is significantly correlated with immune-related gene sets. Moreover, FCGBP also contributes to M2 macrophage polarization by acting as an oncogene in ovarian cancer [24]. Overexpressed HOXB3 in various cancers promotes tumor progression [25–27]. The mRNA and protein expressions of HOXB3 are significantly upregulated in primary prostate cancer tissues compared with the adjacent normal prostate tissues. Furthermore, overexpression of HOXB3 increases prostate cancer proliferation through transcriptional activation of cell division cycle associated 3 [28]. Multivariate analysis demonstrated that HOXB3 (HR  =  1.09, 95% CI 1.01–1.17, p  =  0.027) overexpression is closely associated with shorter PFS, and HOXB3 overexpression decreased the sensitivity of ovarian cancer to cisplatin and attenuates the generation of cisplatin-induced ROS [29]. CLDN10 is upregulated in hepatocellular carcinoma (HCC) tissues, and patients with higher CLDN10 protein level prone to develop a poor prognosis [30]. CLDN10 also promotes papillary thyroid cancer cell growth and invasion, similarly, patients in high-expressed CLDN10 group show a worse prognosis [31]. In ovarian cancer, a low expression level of CLDN10 is associated with a less favorable prognosis [32]. As for TYMSOS, there is no report on its relationship with tumor or related mechanism, which therefore requires further study. To the best of our knowledge, the current study is the first to correlate these genes with the prognosis and clinical characteristics of ovarian cancer, providing evidence for further study of its molecular mechanisms.

The results of this study showed that memory B cell and eosinophil cell infiltration was significantly increased in the high-risk score group (C1), while activated CD4 T cell, activated CD8 T cell, type 1 T helper cell, type 2 T helper cell, and CD56dim natural killer cell infiltration were significantly increased in the low-risk score group (C2). Study considered eosinophil infiltration as indicative of an unfavorable prognosis in colorectal, breast and prostate cancers [33], and this may help explain the increased eosinophil cell infiltration observed in the high-risk score group. In contrast, a local increase in the density of CD8  +  T-cell infiltration in tumors is a marker of a favorable prognosis, which may explain the increased CD8 + T-cell infiltration observed in the lower risk score group with a better prognosis. The infiltration of these cells reflects the immune microenvironment of breast cancer and may also predict prognosis.

This study applied a combination of multiple sets, multiple data sets, and multiple analyses to examine the robustness of our results. However, there are still certain limitations in this study and further improvement is needed. Although this study included two study cohorts, our findings should be confirmed in a separate cohort. Further basic experiments are indispensable in revealing the molecular mechanism of m5C regulators in ovarian cancer progression and in further testing test the predictive efficacy of this feature for clinical application.

## Conclusion

In conclusion, our study found that 4 m5C-related genes were significantly associated with prognosis of ovarian cancer patients, therefore, the 4-gene signature with clinicopathological characteristics could be a useful biomarker for predicitng the prognosis of ovarian cancer.

## Methods

### Ovarian cancer dataset acquisition and process

All ovarian cancer mRNA expression profiles and corresponding clinical data used in our study were downloaded from the TCGA data portal (http://gdc-portal.nci.nih.gov/) and gene expression omnibus (GEO) (GSE30161 dataset). We collected clinical data of 347 cases of ovarian cancer from TCGA. The inclusion criteria were specifically as follows: ovarian cancer samples with clinical follow-up information, transcriptome data, and clinical stage III and IV. In GSE30161 dataset, 58 ovarian cancer samples with clinical information were retrieved. The clinical statistics of the samples are shown in Table 1.

**Table 1:** Clinical information of the samples

Clinical features	TCGA-OV	GSE30161
OS		
0	125	22
1	222	36
Stage		
III	290	15
IV	57	43
Grade		
G1	1	
G2	35	
G3	302	
G4	1	
GX	8	
Stage		
III	290	15
IV	57	43
Gender		
≤ 60	192	25
> 60	155	33

### Identification of molecular subtypes

Based on the expression of 13 m5C regulators, 347 ovarian cancer samples were clustered by the non-negative matrix clustering algorithm (NMF), which was based on the standard “brunet” and performed 50 iterations. The number of clusters k was set from 2 to 10.

### Immunological infiltration analysis between molecular subtypes

MCPcounter was used to evaluate the scores of 10 immune cells. The scores of 28 immune cells were determined by SSGSEA method of GSVA package [34].

### Identification of differentially expressed genes

Limma packets [35] were used to calculate the differentially expressed genes between tumor and normal in the TCGA dataset, according to the threshold of FDR  <  0.05 and |log2FC|  >  1.5.

### Sample grouping

Firstly, 347 tumor samples from the TCGA data set were divided into training set and validation set. To avoid random allocation bias that may affect the stability of subsequent modeling, all the samples were put back into random groups for 100 times in advance. Here, grouping sampling was conducted in accordance with the ratio of training set: validation set  =  1:1. The two groups were similar in terms of stage, grade, and OS. There are 174 samples in the final TCGA training set and 173 samples in the TCGA test set (Table 2). Chi-square test was used to test the training set and test set samples, and there was no significant difference between groups (p  >  0.05).

**Table 2:** Classification information of TCGA sample

Clinical Features	TCGA-train	TCGA-test	P
OS			
0	59	66	1
1	115	107	
Stage			
III	148	142	1
IV	26	31	
Grade			
G1	0	1	0.09157819
G2	18	17	
G3	152	150	
G4	0	1	

### Cox risk analysis for univariate survival

Univariate cox proportional risk regression models were performed using the R package survival coxph function [36] for each differential mRNA and survival data in the TCGA training set data, with p  <  0.05 as the threshold value.

### Model construction

Based on the genes obtained from the univariate cox analysis, genes were further compressed by LASSO cox regression using the R package glmnet to reduce the number of genes in the risk model. In addition, stepwise regression applied the AIC red pool information criterion, which takes into account the statistical fit of the model and the number of parameters. The stepAIC method in the MASS package starts with the most complex model and sequentially removes a variable to reduce the AIC, with a smaller value indicating a more efficient model. Combining the expression of each prognosis-related genes, we developed an independent prognosis model. The RiskScore was calculated using the following formula:

RiskScore  =  0.1452663*FCGBP  +  0.1300001*HOXB − 0.1675289*TYMSO − 0.1130089*CLDN10.

### Evaluation of the Riskcore in TCGA cohort and GEO dataset

According to our prognostic model, each patient in the TCGA test cohort, the entire TCGA dataset and GSE30161 dataset was assigned a risk score. In each cohort, we used the median risk score as a cutoff to classify lung adenocarcinoma patients into high-risk and low-risk groups. Survival curves were plotted using the Kaplan–Meier (KM) method, and log-rank tests were performed to assess the difference in survival between the high-risk and low-risk groups. The receiver operating characteristic curve (ROC) was established using the “timeROC” software package [37], and the area under the curve (AUC) value was calculated to evaluate the specificity and sensitivity of the model. In addition, a prognostic nomogram based on the cox proportional hazards regression model was performed to visualize the relationship between individual predictors and survival of lung cancer patients by using the “rms” package of R software [38]. The performance of the prognostic line graph was evaluated by C index and calibration curves.

To further assess whether our model could be used as an independent prognostic factor, age, sex, stage, T, M, and N were regarded as independent variables. Then univariate cox regression analyses and multivariate cox regression analyses were performed to detect the changes in survival time and survival outcomes.

## Supplementary Information

**Additional file 1: Figure S1.** Functional enrichment analysis of differentially expressed genes.

**Additional file 2: Figure S2.** FCGBP and CLDN10 genes are prognostic factors for ovarian cancer.

**Additional file 3: Figure S3.** The prognostic performance of RiskScore in the TCGA dataset.

**Additional file 4: Figure S4.** The prognostic performance of RiskScore in high-grade serous carcinoma and low-grade serous carcinoma patients.

## Data Availability

The datasets used and/or analyzed during the current study are available from the corresponding author on reasonable request.

## Abbreviations

m5C: 5-methylcytosine
TCGA: The cancer genome atlas
GO: Gene ontology
GSEA: Gene set enrichment analysis
LASSO: Least absolute shrinkage and selection operator
ROC: Receiver operating characteristic
