# Supplementary figures and images for: Identification of 5-methylcytosine-related signature for predicting prognosis in ovarian cancer
Source: Biol Res. 2021 Jun 29;54:18. doi: 10.1186/s40659-021-00340-8 (PMC8240302; doi:10.1186/s40659-021-00340-8)

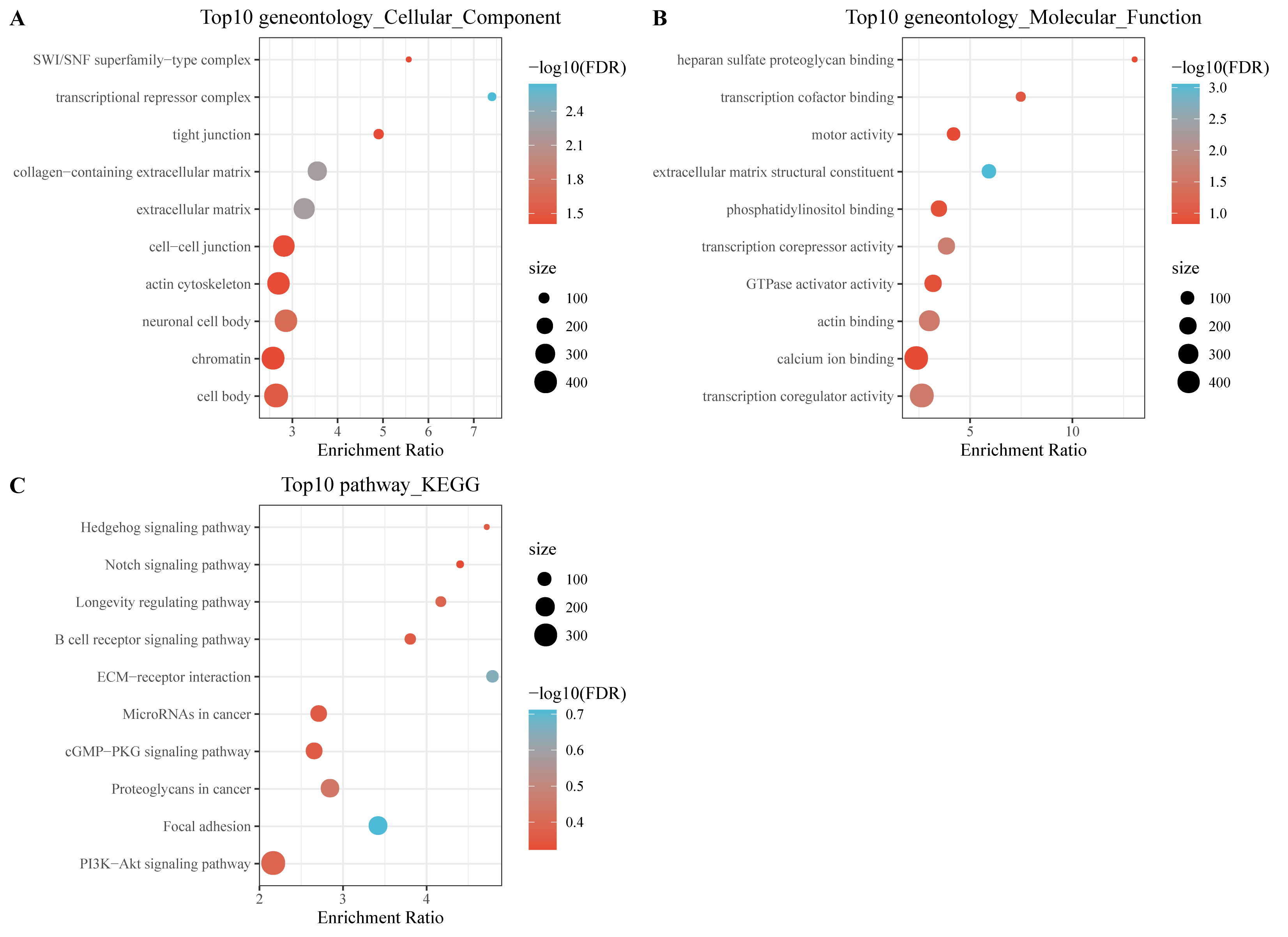

Supplement: Supplementary file 1 — Additional file 1: Figure S1. Functional enrichment analysis of differentially expressed genes. [file 40659_2021_340_MOESM1_ESM.tif]

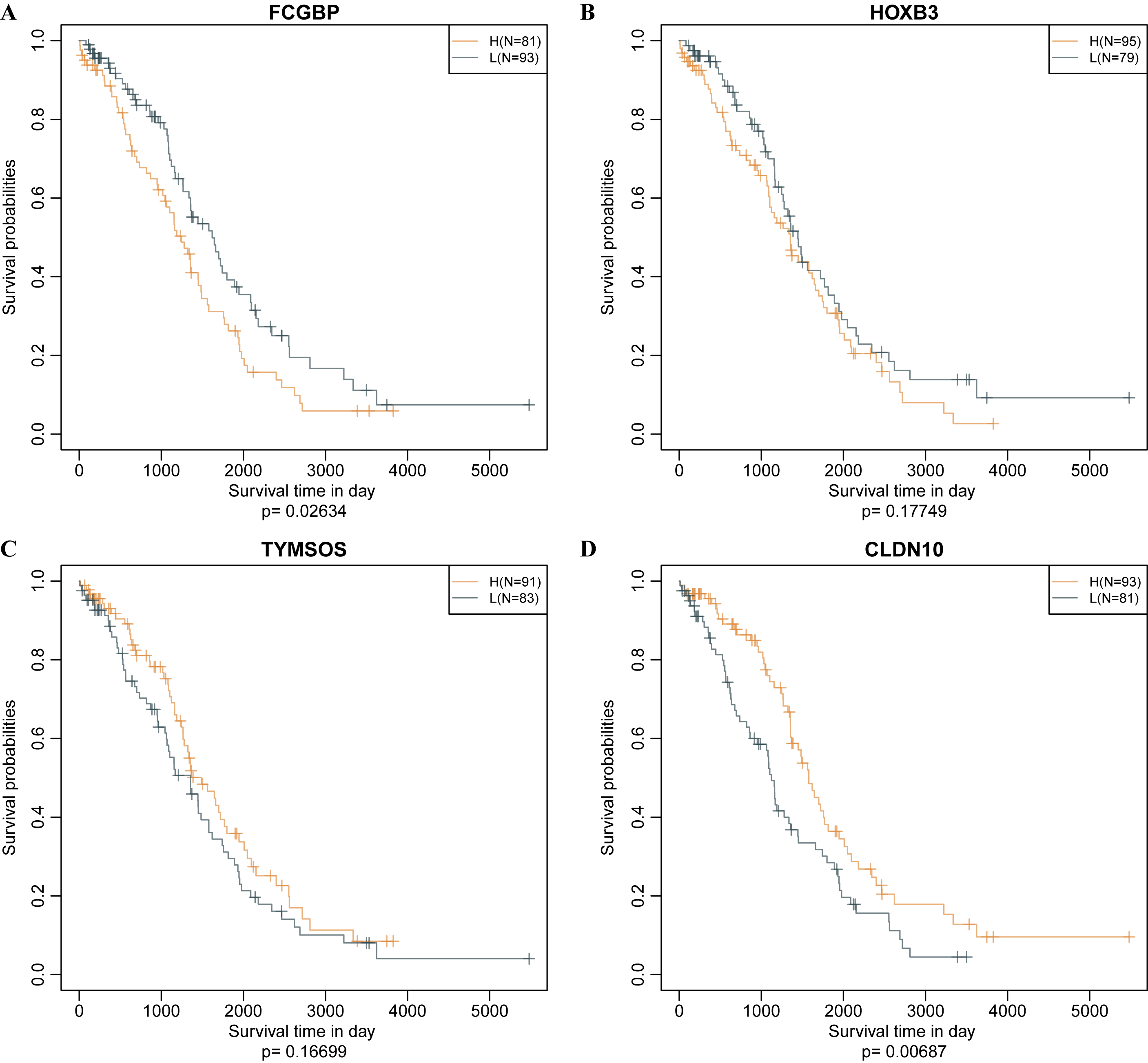

Supplement: Supplementary file 2 — Additional file 2: Figure S2. FCGBP and CLDN10 genes are prognostic factors for ovarian cancer. [file 40659_2021_340_MOESM2_ESM.tif]

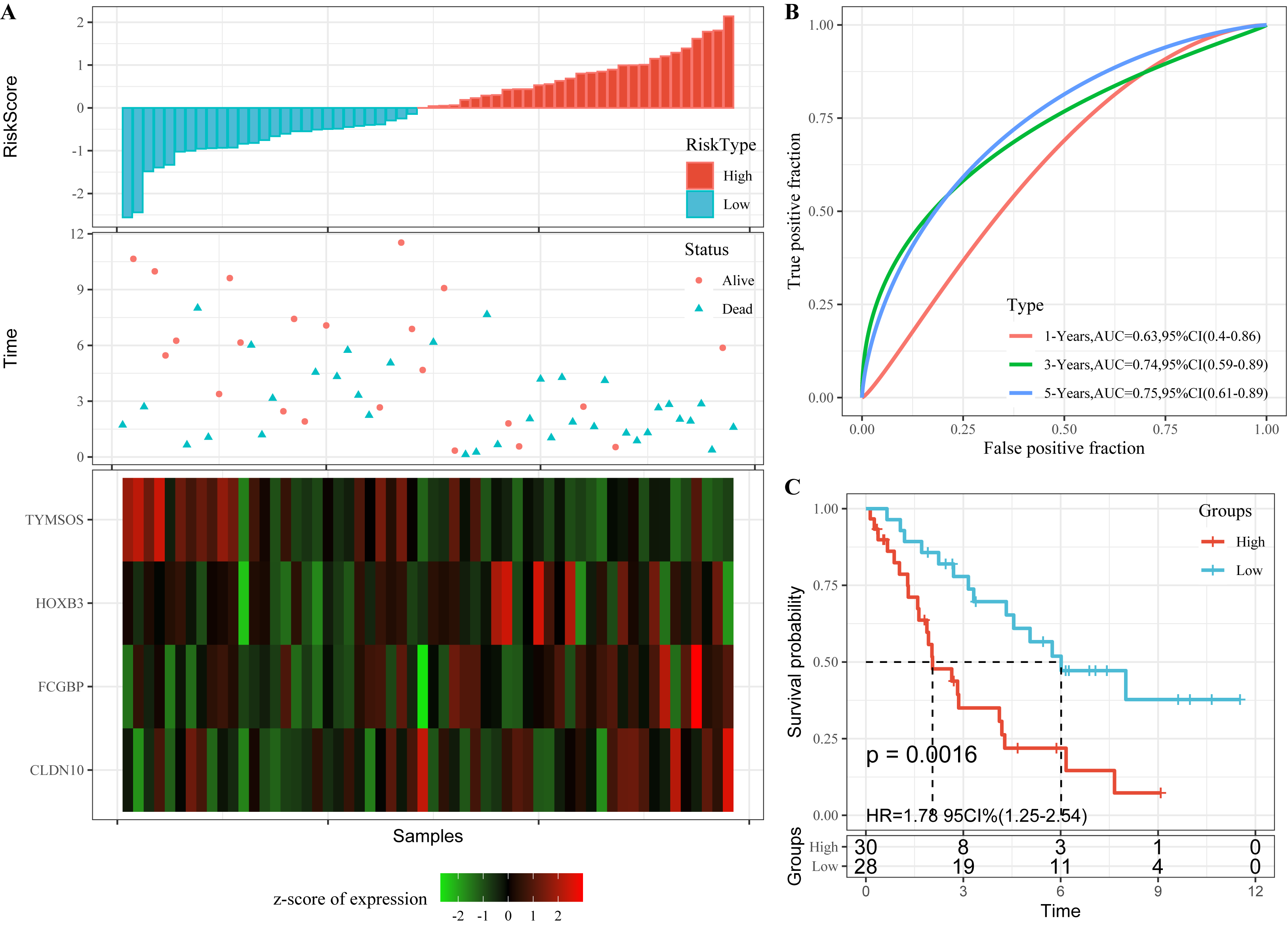

Supplement: Supplementary file 3 — Additional file 3: Figure S3. The prognostic performance of RiskScore in the TCGA dataset. [file 40659_2021_340_MOESM3_ESM.tif]

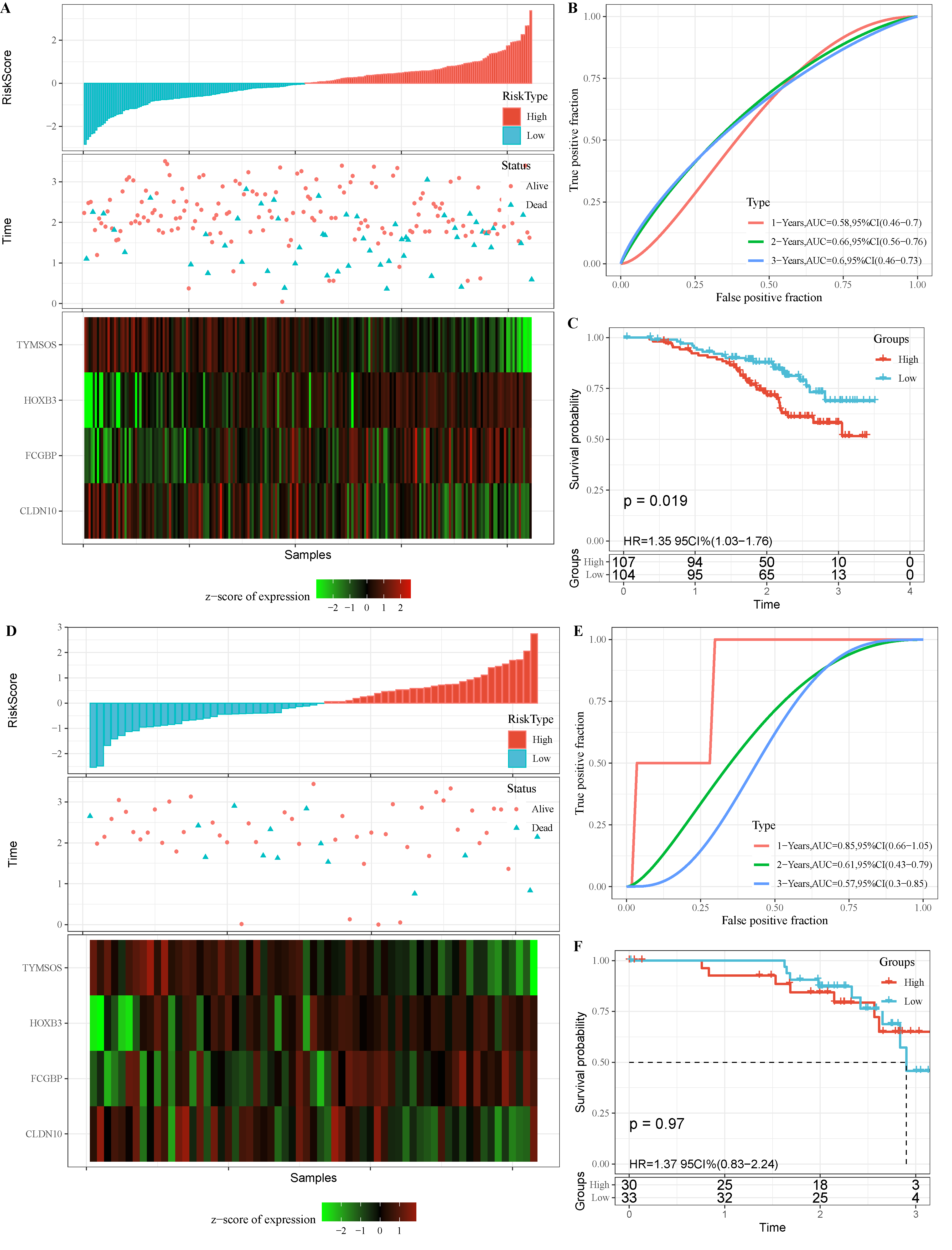

Supplement: Supplementary file 4 — Additional file 4: Figure S4. The prognostic performance of RiskScore in high-grade serous carcinoma and low-grade serous carcinoma patients. [file 40659_2021_340_MOESM4_ESM.tif]
